# Clinical significance of Ankyrin repeat domain 12 expression in colorectal cancer

**DOI:** 10.1186/1756-9966-32-35

**Published:** 2013-05-29

**Authors:** Rui Bai, Dan Li, Zhong Shi, Xuefeng Fang, Weiting Ge, Shu Zheng

**Affiliations:** 1 Cancer Institute, The Second Affiliated Hospital, Zhejiang University School of Medicine, 88 Jiefang Road, Hangzhou, Zhejiang, China; 2 Key Laboratory of Cancer Prevention and Intervention, China National Ministry of Education, Hangzhou, Zhejiang, China; 3 Department of Medical Oncology, Hangzhou First People’s Hospital, Hangzhou Cancer Hospital, Hangzhou, Zhejiang, China; 4 Department of Medical Oncology, The Second Affiliated Hospital Zhejiang University School of Medicine, Hangzhou, Zhejiang, China

**Keywords:** Ankyrin repeat domain 12 (ANKRD12), Colorectal cancer, Metastasis, Prognostic factor

## Abstract

**Background:**

Ankyrin repeat domain 12 (ANKRD12), is encoding a 224 kDa nuclear protein and most conserved at its N-terminal ankyrin repeats region and the C-terminal co-activator interacting domain. The aim of this study was to investigate the ANKRD12 mRNA expression in colorectal cancer (CRC) tumor tissues and the normal adjacent mucosa and its potential relevance to clinicopathological characteristics and prognosis.

**Methods:**

Surgical specimens of tumor tissues (n = 68) and adjacent normal mucosa (n = 51) were obtained from CRC patients. The ANKRD12 mRNA expression was measured by quantitative real time reverse transcriptase polymerase chain reaction. The relationship between ANKRD12 mRNA expression and clinicopathological features was analyzed by appropriate statistics. Kaplan–Meier analysis and Cox proportional hazards regression models were used to investigate the correlation between ANKRD12 expression and prognosis of CRC patients.

**Results:**

The relative mRNA expression of ANKRD12 were significantly lower in CRC tumor tissues than in the normal adjacent mucosa (P < 0.001), and the cases with low ANKRD12 expression showed a higher frequency of liver metastasis (P = 0.015). Kaplan–Meier analysis indicated that patients (CRC without liver metastasis) with low ANKRD12 expression had poor overall survival (P = 0.041). Multivariate analysis showed that low ANKRD12 expression was an independent predictor of overall survival.

**Conclusion:**

This study revealed that ANKRD12 mRNA were down regulated in CRC tumor tissues and low ANKRD12 expression was correlated with liver metastasis and poor survival of CRC patients.

## Background

Colorectal cancer (CRC) is one of the leading causes of cancer mortality in the world. An estimated 143,460 new cases of CRC occurred in 2012 and 51,690 people have died from CRC during the same year [1]. In China, the incidence of CRC has been increasing continually in the most recent years. Although we have made considerable advances in diagnosis and adjuvant therapy of CRC, the overall survival rate of CRC patients has not been improved markedly. Therefore, there is an urgent need for better understanding of CRC pathogenesis which may lead to more effective treatment strategies.

Ankyrin repeats-containing cofactor (ANCO) proteins contain both ankyrin repeat and transcriptional repression/activation domains [2-4], including cyclin-dependent kinase inhibitors, transcription factors and cytoskeleton organizers [5]. These proteins were first identified in two yeast cell-cycle regulators, Swi6P and CDC10P, and in the Notch and LIN-12 development regulators [6]. ANCO proteins can inhibit the transcriptional activity of nuclear receptors through the recruitment of histone deacetylases (HDACs) [2]. Ankyrin repeat domain 11 (ANKRD11), also called ANCO-1, is a member of the ankyrin repeats-containing cofactor family. ANKRD11 was identified from a yeast two-hybrid screen as a ras-related C3 botulinum toxin substrate 3(RAC3)-interacting protein [2], and was thought to recruit HDACs to the p160 co-activator to repress transcriptional activation by nuclear receptors [7]. The N-terminal domain of the ANKRD11 was also reported as a nasopharyngeal carcinoma-susceptibility protein LZ16, and the central region of ANKRD11 was isolated as a tumor antigen present in childhood medulloblastoma [8]. The ANKRD11 gene locus is located within the 16q24.3 breast cancer loss of heterozygosity (LOH) region [9]. LOH of chromosome 16q of breast cancer results in the simultaneous loss or reduction in activity of several tumor suppressor genes, and this contributes to the early stages of tumorigenesis. Recently research also showed that ANKRD11 was a p53 coactivator and involved in a regulatory feedback loop with p53 in breast cancer [10]. All of these showed ANKRD11 is a putative tumour-suppressor gene.

Ankyrin repeat domain 12 (ANKRD12), also called ANCO-2, is encoding a 224 kDa nuclear protein and most conserved at its N-terminal ankyrin repeats region and the C-terminal co-activator-interacting domain [7]. The similarity between ANKRD12 and ANKRD11 is most striking at the N-terminal and C-terminal domains (67 and 81%, respectively) [2]. ANKRD12 also interacts with p160 coactivators in GST pull-down assay suggesting that ANKRD12 and ANKRD11 may be functionally related [2]. However, the precise role of ANKRD12 in transcriptional regulation and tumor process is less clear.

In our study, the mRNA expression of ANKRD12 was measured in cancer tissue and adjacent normal mucosa of CRC by quantitative real time reverse transcriptase polymerase chain reaction (qRT-PCR).We studied the correlation between the relative expression of ANKRD12 and clinicopathological features to evaluate its clinical significance. Additionally, we assessed the influence of ANKRD12 expression on the outcomes of CRC patients.

## Materials and methods

### Patient and tissue samples

Tumor samples (n = 68) and adjacent normal mucosa (n = 51) were obtained from CRC patients undergoing primary tumor resection at the Second Affiliated Hospital of Zhejiang University during the period between 2001 and 2007. The ethics committee of Zhejiang University approved the study. The tissue samples were snap-frozen in liquid nitrogen and stored at −80°C until used. Patients were evaluated at 3-month intervals for the first year after surgery and at 6-month intervals after. The follow-up was standard all patients. All patients were followed up by the Cancer Research Institute until June 2012, and the data concerning cancer recurrence and patient survival were collected. The histopathology of each specimen was reviewed on the H&E-stained tissue section to confirm diagnosis and tumor content at least 70% of tumor cells in the tissue sample.

### Isolation of RNA and quantitative reverse transcription PCR Analysis

Total mRNA was isolated from frozen samples using the NucleoSpin RNA II Kit (Macherey-Nagel, GA). Each mRNA sample (5 μg) was reverse transcribed using the RT-PCR Kit (Promega). Transcript level of ANKRD12 was determined by quantitative reverse transcription PCR (qRT-PCR) using the Applied Biosystems StepOne Real-Time PCR System (Applied Biosystems, Carlsbad, CA). qRT-PCR primers were ANKRD12 5′- TTTTGCGAGTTCATTACAGAGC -3′and 5′- AATTGTCTTGCATTAAAGCGATC -3′, *β–actin* 5′-TTCCAGCCTTCCTTCCTGGG-3′ and 5′-TTGCGCTCAGGAGGAG CAAT-3′. Human β–actin was amplified as an endogenous control. The qRT-PCR reactions were carried out in a total volume of 20 μl per well containing SYBR master mix reagent kit (Applied Biosystems, Carlsbad, CA) in triplicate. The relative gene expression was calculated by the equation 2^-ΔΔCT^.

### Statistical analysis

qRT-PCR data were calculated with StepOne Software v2.1 (Applied Biosystems, Carlsbad, CA). Measurement data were analyzed by Student’s t-test, while categorical data were analyzed by chi-square test. The postoperative survival rate was analyzed with Kaplan–Meier method, and the log-rank test was used to assess the significance of differences between survival curves. The statistical analyses were performed using SPSS 16.0 software (SPSS, Chicago, IL, USA). All differences were considered statistically significant if the *P* value was <0.05.

## Results

### ANKRD12 mRNA expression in colorectal cancer and normal adjacent mucosa

**Figure 1:**
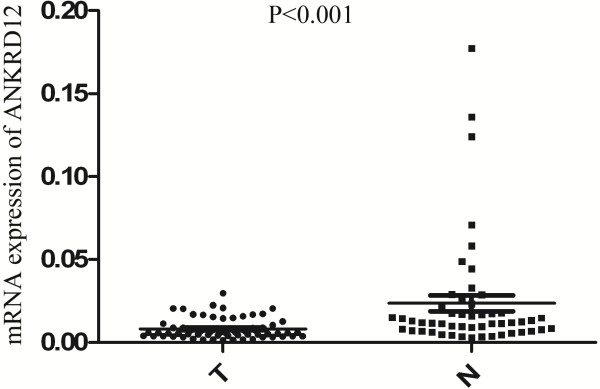
**Quantitative reverse transcription-PCR showed mRNA expression of ANKRD12 in CRC tumor tissues (T) and adjacent normal mucosa (N).** ANKRD12 expression levels were lower in tumor tissue than in normal adjacent mucosa (p < 0.001, Student’s t test).

By qRT-PCR, we showed that ANKRD12 expression in cancer tissues were significantly lower ( P < 0.001) than those in the normal adjacent mucosa (Figure 1).

### Relationship between ANKRD12 mRNA expression and clinicopathological features

The mRNA expression of the ANKRD12 was categorized as low or high in relation to the median value. The experimental samples were divided into two groups [the high ANKRD12 expression group (n = 34) and the low ANKRD12 expression group (n = 34)] to investigate ANKRD12 mRNA expression in association with clinicopathologic variables (Table 1). The ANKRD12 mRNA expression was not related to age, gender, histological type, depth of invasion(T), lymph node metastasis, tumor location. However, the incidence in liver metastasis was significantly higher (P = 0.015) in the low expression group (14 of 34, 41.2%) than in the high expression group (5 of 34, 14.7%), and the incidence of cancer death was significantly higher (P = 0.015) in the low expression group (22 of 34, 64.7%) than in the high expression group (12 of 34, 35.3%).

**Table 1:** Clinicopathologic variables and ANKRD12 mRNA expression in 68 colorectal cancers

**Variables**	**Expression**		***P *value**
	**ANKRD12 high**	**ANKRD12 low**	
	**(n = 34)**	**(n = 34)**	
Age	58.0 ± 15.0	61.6 ± 14.1	0.309
Sex			0.215
Male	18	23	
Female	16	11	
Histological type			0.793
Well, Moderate	23	24	
Poor and others	11	10	
Depth of invasion			0.380
T1,2,3	25	28	
T4	9	6	
Location			0.086
Colon	23	16	
Rectum	11	18	
Lymph node metastasis			0.209
Absent	15	10	
Present	19	24	
Liver metastasis			0.015*
Absent	29	20	
Present	5	14	
Cancer-related death			0.015*
Alive	22	12	
Death	12	22	

*n* Number of patients, * <0.05.

### ANKRD12 mRNA expression and prognosis of CRC patients

**Figure 2:**
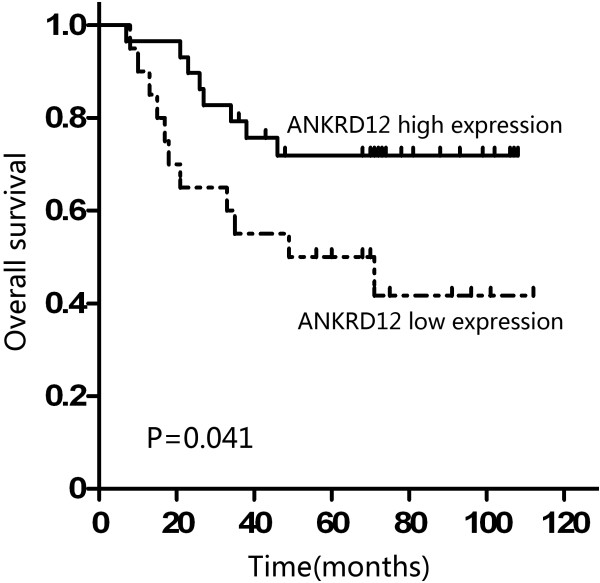
**Kaplan-Meier survival curves of CRC patients without liver metastasis according to the status of ANKRD12 expression.** Patients with low ANKRD12 mRNA expression showed significantly poorer prognosis than those with high ANKRD12 mRNA expression (P = 0.041, log-rank test).

Overall survival curves were plotted according to ANKRD12 mRNA expression by the Kaplan–Meier method. In the study group of CRC without liver metastasis (49 patients), the overall survival rate was significantly lower in the patients with low ANKRD12 mRNA expression than that in those with high expression (P = 0.041; Figure 2).

Univariate analysis with Cox proportional hazards model identified four prognostic factors: location, lymph node metastasis, liver metastasis, and ANKRD12 expression. The other clinicopathological features, such as age, gender, histological type and depth of invasion were not statistically significant prognosis factors (Table 2).

**Table 2:** Univariate analysis of clinicopathological factors for overall survival

	**n**	**Hazard ratio**	**95% CI**	***P *value**
Age (years)				
≤60	36	1		
>60	32	1.811	0.905-3.624	0.093
Sex				
Male	41	1		
Female	27	1.077	0.544-2.134	0.831
Histological type				
Well, moderate	47	1		
Poor and others	21	1.627	0.813-3.256	0.169
Depth of invasion				
T1,2,3	53	1		
T4	15	0.691	0.300-1.589	0.385
Location				
Colon	39	1		
Rectum	29	1.978	1.005-3.891	0.048*
Lymph node metastasis				
Absent	25	1		
Present	43	2.432	1.098-5.385	0.028*
Liver metastasis				
Absent	49	1		
Present	19	9.764	4.590-20.768	0.000*
ANKRD12				
High	34	1		
Low	34	2.566	1.267-5.201	0.009*

*n* Number of patients, *CI* confidence interval, * <0.05.

Table 3 shows the result of multivariate analysis of in the final model, which included age, histological type, depth of invasion, location, lymph node metastasis and ANKRD12 expression. In this model, the variable of low ANKRD12 expression was an independent prognostic predictor for CRC patients (HR, 2.772; 95% CI, 1.065-7.211; P = 0.037; Table 3). Of the patients that were entered in the multivariate analysis, patients with liver metastasis were excluded because the presence of liver metastasis was a strong prognostic factor and was associated with low expression of ANKRD12.

**Table 3:** Multivariate analysis of clinicopathological factors for overall survival (CRC without liver metastasis)

	**Hazard ratio**	**95% CI**	***P *value**
Age (>60/≤60)	0.574	0.208-1.441	0.222
Histological type (Poor and others/ Well, Moderate)	1.442	0.542-3.836	0.464
Depth of invasion (T4/ T1,2,3)	1.478	0.564-3.873	0.426
Location (Rectum/Colon)	2.002	0.770-5.203	0.154
Lymph node metastasis (present/absent)	1.884	0.671-5.295	0.229
ANKRD12 (low/high)	2.772	1.065-7.211	0.037*

*CI* confidence interval, * <0.05.

## Discussion

Gene expression regulated by steroid/nuclear hormone receptors (NRs) is crucial in many physiological processes. The activity of NRs is first regulated by ligands [11], as binding of cognate ligands triggers a conformational change that causes receptor activation [12]. Upon ligand binding, co-repressors are released from the receptor, and co-activators are recruited to the activated receptor [13]. Ankyrin repeats-containing cofactor (ANCO) proteins are a family of unique transcriptional co-regulators with dual properties: they interact with both the co-activators and the co-repressors [2].

Ankyrin repeat domain 11 (ANKRD11), also called ANCO-1, is located within the 16q24.3 breast cancer loss of heterozygosity (LOH) region [9] and was a p53 coactivator in breast cancer [10], implying a putative tumour-suppressor role. Ankyrin repeat domain 12 (ANKRD12), also called ANCO-2, is highly related to ANKRD11, especially at the ankyrin repeats and C-terminal domain. However, the clinical significance of ANKRD12 expression in cancer remains unclear. In the present study, we confirmed that ANKRD12 was lower in colorectal cancer tissues than in their normal adjacent mucosa and the cases whose tumors had low ANKRD12 expression showed a higher frequency of liver metastasis, strongly suggesting that ANKRD12 might be involved in the carcinogenesis, development and metastasis of CRC. More importantly, we proved that ANKRD12 expression was significantly associated with overall survival of CRC patients. In support of this, Kaplan–Meier analysis of overall survival showed that patients whose tumors had lower ANKRD12 expression tend to have a significantly worse overall survival, indicating that low ANKRD12 level is a marker of poor prognosis for CRC patients. Moreover, Cox proportional hazards model showed that low ANKRD12 expression was an independent prognostic predictor for CRC patients. Therefore, ANKRD12 could constitute a molecular prognostic marker for CRC patients, identifying who are more likely to have higher risk of death and need receive a more aggressive treatment.

The precise molecular mechanisms behind the altered expression of ANKRD12 in colorectal cancer are unclear. To our knowledge, this is the first report to describe the significance of ANKRD12 to clinical stage, lymph node and liver metastases, and prognosis of CRC patients. ANKRD12 binds to alteration/deficiency in activation 3(ADA3) through its C-terminal domain and inhibits ADA3-mediated transcriptional co-activation on NRs [7]. ADA3 is a component of the human P/CAF acetyltransferase complex which is thought to link co-activators to histone acetylation and basal transcription machinery [14]. Gene expression regulated by NRs, therefore ANKRD12 may regulate some important gene expression by inhibiting ADA3-mediated transcriptional co-activation on NRs. Recently, ADA3 is also identified as a p53-binding protein [15-17], as well as causing p53 acetylation [18]. In mammalian cells, overexpression of ADA3 increased p53 levels [16]. P53 was identified as a tumor suppressor protein and is the most commonly mutated gene in human cancers [19-21]. However, ANKRD12 has little or no effect to promote p53 activation [7]. So we speculated that the effects of ANKRD12 in tumor development or progression might, through binding to ADA3 co-activators, increasing p53 levels and inhibit tumor development or progression. Additional studies to investigate the real molecular mechanisms of altered expression of ANKRD12 in the development or progression of CRC are essential.

## Conclusions

In conclusion, we found that ANKRD12 mRNA were downregulated in CRC tumor tissues and low ANKRD12 mRNA expression correlated with poor overall survival and liver metastasis of CRC patients. These findings suggest that ANKRD12 is a cancer-related gene associated with liver metastasis and a survival predictor of CRC patients.

### Consent

Written informed consent was obtained from the patient for publication of this report and any accompanying images.

## Abbreviations

ANKRD12: Ankyrin repeat domain 12; CRC: Colorectal cancer; ANCO: Ankyrin repeats-containing cofactor; HDACs: Histone deacetylases; ANKRD11: Ankyrin repeat domain; LOH: Loss of heterozygosity; RAC3: Ras-related C3 botulinum toxin substrate 3; qRT-PCR: Quantitative real time reverse transcriptase polymerase chain reaction; NRs: Steroid/nuclear hormone receptors; ADA3: Alteration/deficiency in activation 3.

## Competing interests

The authors declare that they have no competing interests.

## Authors’ contributions

RB, DL and SZ conceived and designed the study, performed the experiments and wrote the paper. ZS and XFF contributed to the writing and to the critical reading of the paper. WTG performed patient collection and clinical data interpretation. All authors read and approved the final manuscript.
